# Genetic and Environmental Contributions to Facial Morphological Variation: A 3D Population-Based Twin Study

**DOI:** 10.1371/journal.pone.0162250

**Published:** 2016-09-01

**Authors:** Jelena Djordjevic, Alexei I. Zhurov, Stephen Richmond, Visigen Consortium

**Affiliations:** Department of Applied Clinical Research and Public Health, School of Dentistry, Cardiff, United Kingdom; NIDCR/NIH, UNITED STATES

## Abstract

**Introduction:**

Facial phenotype is influenced by genes and environment; however, little is known about their relative contributions to normal facial morphology. The aim of this study was to assess the relative genetic and environmental contributions to facial morphological variation using a three-dimensional (3D) population-based approach and the classical twin study design.

**Materials and Methods:**

3D facial images of 1380 female twins from the TwinsUK Registry database were used. All faces were landmarked, by manually placing 37 landmark points, and Procrustes registered. Three groups of traits were extracted and analysed: 19 principal components (uPC) and 23 principal components (sPC), derived from the unscaled and scaled landmark configurations respectively, and 1275 linear distances measured between 51 landmarks (37 manually identified and 14 automatically calculated). The intraclass correlation coefficients, r_MZ_ and r_DZ_, broad-sense heritability (h^2^), common (c^2^) and unique (e^2^) environment contributions were calculated for all traits for the monozygotic (MZ) and dizygotic (DZ) twins.

**Results:**

Heritability of 13 uPC and 17 sPC reached statistical significance, with h^2^ ranging from 38.8% to 78.5% in the former and 30.5% to 84.8% in the latter group. Also, 1222 distances showed evidence of genetic control. Common environment contributed to one PC in both groups and 53 linear distances (4.3%). Unique environment contributed to 17 uPC and 20 sPC and 1245 distances.

**Conclusions:**

Genetic factors can explain more than 70% of the phenotypic facial variation in facial size, nose (width, prominence and height), lips prominence and inter-ocular distance. A few traits have shown potential dominant genetic influence: the prominence and height of the nose, the lower lip prominence in relation to the chin and upper lip philtrum length. Environmental contribution to facial variation seems to be the greatest for the mandibular ramus height and horizontal facial asymmetry.

## Introduction

Facial morphology attracts interest from a variety of scientific disciplines dealing with craniofacial evolution (anthropology) [1], forensic facial reconstruction and identification (forensic science) [2], facial recognition (computer science) [3], predictions of facial ageing, growth and development (clinical orthodontics) [4], and the perception of facial features in social interactions (sociology and psychology) [5]. The common goal of all these disciplines has been a greater understanding of the factors that shape facial morphology in various populations. Human Genome and Phenome projects revolutionized the way scientists have looked at this topic. For a long time, dysmorphic facial characteristics have been the focus of clinical and genetic studies, but the same could not have been said about normal facial variation until recently [6].

Facial morphology is influenced by genetic and environmental factors and their complex interactions that present the greatest challenge in modern biology [7,8]. Traditionally, exploration of genetic variance in humans has been limited mostly to the use of additive effects estimated using pedigree data [9]. The role of genetics in complex traits has been quantified as heritability, e.g. the proportion of the total phenotypic variance explained by additive genetic variance. However, genetic variance of complex traits also includes non-additive variances (dominance and epistasis) [9,10]. With the development of dense panels of single nucleotide polymorphisms (SNPs), the exploration of genetic variation of complex traits is moving to the dissection of genetic variation at individual loci [11,12].

Various environmental influences are also responsible for specific craniofacial shape, including hormones, nutrition, diseases, trauma, surgery, dentofacial orthopaedics, lifestyle factors (e.g. smoking, alcohol, exercise) and oral functions (mastication, respiration, swallowing) [13]. Environmental factors can influence epigenetic mechanisms (acquired and heritable changes in gene function that occur without a change in the DNA sequence) [14]. This can be accomplished through DNA modification (i.e., methylation), histone modification and post-transcriptional silencing by RNA interference. Thus, transcriptional activity of specific genes is controlled at specific points in time in specific organs [15].

Heritability of craniofacial characteristics has been investigated in family (siblings or parents-offspring design) and twin studies (monozygotic, MZ vs. dizygotic twins, DZ) mainly using two-dimensional techniques (anthropometry, photography and lateral skull radiography) [16–23]. The results of these studies are limited mainly due to: 1) a significant loss of information when faces are studied in two dimensions only; 2) risks associated with radiation hazard, 3) issues arising in determining zygosity (it was not always confirmed formally by genetic testing), 4) relatively small samples and a loss of statistical power. Recent three-dimensional (3D) techniques (such as laser scanning, photogrammetry and magnetic resonance imaging) have enabled non-invasive data collection and a more comprehensive description of facial features [24–27].

Over the last decade, a few studies have investigated 3D facial characteristics of twins [28–31]. Using facial colour maps of twins (depicting the deviation between faces), Naini and Moss [29] found that the triangular area encompassing the orbital rims, intercanthal area, and the nose is genetically driven in British twins; MZ twins showed similarities in the shape of the eyebrows, bridge of the nose and infraorbital ridges, whereas other parts of the face such as cheeks, chin, and lips showed significant variation [31]. Similar findings were reported in the preliminary study performed on American twins [32]. These 3D studies dealt with small, convenient samples that were not representative of the respective populations. In addition, there was no robust estimation of heritability. Therefore, our current knowledge on heritability of craniofacial traits mainly stems from two-dimensional studies with some conflicting results on heritability estimates of horizontal and vertical skeletal parameters.

The aim of the present study was to assess genetic and environmental contributions to 3D facial soft tissue morphological variation within the framework of the classical twin study design [33]. The objectives were to: 1) evaluate additive and non-additive genetic effects and 2) evaluate the influence of common and unique environment on facial morphological traits of British adult female twins.

## Materials and methods

### Sample

The sample comes from the UK Adult Twin Registry (Twins UK, www.twinsuk.ac.uk), a population-based study of twins with ongoing longitudinal data collection [34]. At the time the study was conducted, the Registry database contained 3D facial images of 1521 twins aged 16.5 to 87.3 years (98.6% females), obtained using a non-invasive stereophotogrammetry device (3dMDface system, 3dMD Inc., Atlanta, GA, USA) proven to be valid and reliable [35–37]. Facial images of 1380 female participants aged between 23.6 and 86.5 years (mean 58.8, SD 10.5) were used in this study. There were 263 pairs of MZ twins, 341 pairs of DZ twins and 172 unpaired twins (75 MZ and 97 DZ): 31 individuals whose facial image was not taken and 141 individuals who were excluded for one of the following reasons: 1) zygosity was initially assessed by a ‘peas in a pod’ questionnaire but not confirmed via subsequent genotyping or genome-wide association studies (n = 19); 2) male participants (n = 15); 3) image quality was not good enough to identify 37 facial landmarks (n = 99); 6) facial expression was not neutral (n = 3); 7) head posture was not natural (n = 3); 8) mouth open and/or eyes closed (n = 2). Ethical approval for the study was obtained from the Health Research Authority UK (more specifically from its National Research Ethics Service Committee in London, Ref: EC04/015). Written consent was obtained from all participants.

### Data collection

During the taking of 3D facial images, the participants were instructed to do the following: sit up straight in front of the device and look at the distance; to keep the head in the natural head posture; to maintain neutral facial expression with mouth closed, lips at rest (if competent) and eyes open; and to be still. In addition, they were asked to take off their glasses and tie up their hair. Image acquisition took approximately 3 minutes per person (1.5 ms shot followed by automated transfer, processing and triangulation of the data). The quality of the images was checked using the proprietary software 3dMD Patient. The images were saved as TSB files (a 3dMD Patient format) and converted to OBJ format for further analysis with Rapidform 2006 (INUS Technology Inc., Seoul, South Korea).

Processing, normalising and landmarking of facial images was performed using in-house developed subroutines for Rapidform 2006 [38]. First, the background objects (if any were captured), pieces of clothes, hair and most of the neck were removed using a freehand cutting tool. The area of the neck approximately 2 cm under the mandibular body and ramus was left to enable correct identification of two landmarks: ‘gnathion’ and ‘gonion’. Secondly, the faces were normalized by fitting them into the same reference frame. The origin of this reference system (with the coordinates x = 0, y = 0, z = 0) was the point halfway between the inner canthi of the eyes. Coronal plane (xy) was determined by the cylinder that fitted all data points of structure made from the original face and its mirror reflection. Sagittal plane (yz) was identified as the symmetry plane of that structure. Transverse plane (xz) was perpendicular to the previous two planes and connected the inner canthi of the eyes [38]. The normalization procedure was performed to facilitate landmarking and construct average faces [27].

**Fig 1:**
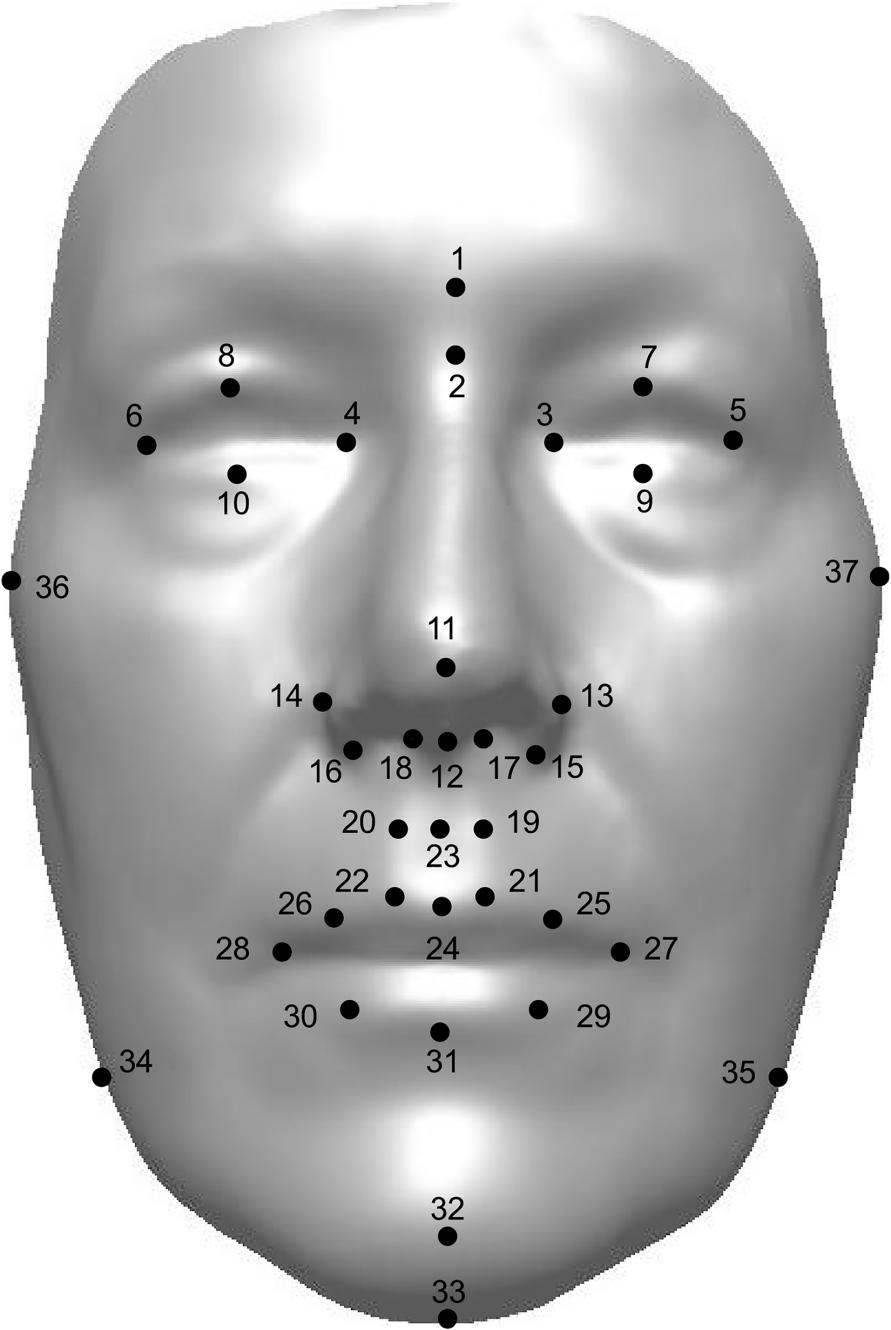
Thirty-seven anthropometric landmarks (14 bilateral and 9 mid-sagittal) used in the study. 1) glabella (g); 2) nasion (n); 3) endocanthion left (enl); 4) endocanthion right (enr); 5) exocanthion left (exl); 6) exocanthion right (exr); 7) palpebrale superius left (psl); 8) palpebrale superius right (psr); 9) palpebrale inferius left (pil); 10) palpebrale inferius right (pir); 11) pronasale (prn); 12) subnasale (sn); 13) alare left (all); 14) alare right (alr); 15) subalare left (sbal); 16) subalare right (sbar); 17) philtrum-nasale left (phnl); 18) philtrum-nasale right (phnr); 19) mid-philtrum left (mphl); 20) mid-philtrum right (mphr); 21) crista philtri left (cphl); 22) crista philtri right (cphr); 23) deepest point of the philtrum (dpc); 24) labiale superius (ls); 25) mid-upper lip left (mull); 26) mid-upper lip right (mulr); 27) cheilion left (chl); 28) cheilion right (chr); 29) mid-lower lip left (mlll); 30) mid-lower lip right (mllr); 31) labiale inferius (li); 32) pogonion (pg); 33) gnathion (gn); 34) gonion right (gor); 35) gonion left (gol); 36) zygion right (zyr); 37) zygion left (zyl).

Stereophotogrammetry enables capturing of thousands of points across the facial surface that contribute to facial variation [24–27]. In this study, the analyses were limited to landmark configurations and linear distances based on 37 anthropometric landmarks (14 bilateral and 9 mid-sagittal). The definitions of eight mid-sagittal and 10 bilateral landmarks were identical to a widely-known anthropometric definitions by Farkas [39]. One mid-sagittal and four bilateral landmarks were newly defined by the authors of the study (Fig 1, Table 1). All landmarks were manually identified on all the images by one operator. For every participant, 111 x, y and z coordinates were saved.

**Table 1:** Definitions of anthropometric landmarks identified on 3D facial images.

Mid-sagittal landmarks (abbreviation)	Definitions
Glabella (g)	The most prominent midpoint between the eyebrows
Nasion (n)	Midline point between the nasal root and nasofrontal suture, above the line that connects the two inner canthi
Pronasale (prn)	The most protruded point of the apex nasi
Subnasale (sn)	The midpoint of the angle at the columella base where the lower border of the nasal septum and the surface of the upper lip meet
The deepest point of the philtrum (dpc)*	The deepest point of the philtrum of the upper lip assessed from the profile view
Labiale superius (ls)	The midpoint of the upper vermilion line
Labiale inferius (li)	The midpoint of the lower vermilion line
Pogonion (pg)	The most prominent midpoint of the chin
Gnathion (gn)	The lowest median point on the lower border of the mandible
**Bilateral landmarks (abbreviation)**	
Endocanthion (en)	The point at the inner commissure of the eye fissure
Exocanthion (ex)	The point at the outer commissure of the eye fissure
Palpebrale superius (ps)	The highest point in the midportion of the free margin of each upper eyelid
Palpebrale inferius (pi)	The lowest point in the midportion of the free margin of each lower eyelid
Alare (al)	The most lateral point on each alar contour
Subalare (sba)	Labial insertion of the alar base
Philtrum-nasale (phn)*	Lateral point at the intersection of the columella base and the philtrum
Mid-philtrum (mph)*	The midpoint of the philtrum (between philtrum-nasale and crista philtri)
Crista philtri (cph)	The point on each elevated margin of the philtrum, just above the vermilion line
Mid-upper lip (mul)*	The midpoint between crista philtri and cheilion on the vermilion border of the upper lip
Cheilion (ch)	The point at each labial commissure
Mid-lower lip (mll)*	The midpoint between cheilion and labiale inferius on the vermilion border of the lower lip
Gonion (go)	The most lateral point on the mandibular angle
Zygion (zy)	The most lateral point of each zygomatic arch

The majority of the landmarks were identified according to the definitions given by Farkas [39]. The landmarks marked with an asterisk (*) were defined by the authors of the study.

### Facial parameters

The first step in obtaining facial parameters for the analyses was the generalized Procrustes registration of landmark configurations. It was performed on the sample of 1380 participants (263 pairs of MZ twins, 341 pairs of DZ twins and 172 unpaired twins) using the R software and programming language [40]. Since both facial shape and size variations were considered important for the study, two registrations were done: without scaling (taking into consideration both facial size and shape) and with scaling (preserving only information about facial shape) [41].

The second step was to import the Procrustes registered coordinates into SPSS (v.20.0, SPSS Inc, Chicago, Illinois, USA) and perform two Principal Component Analyses: the first one was done on the unscaled dataset and the results (principal components) obtained using the covariance matrix and the second one was done on the scaled dataset and the results based on the correlation matrix [42–44]. The principal components were further validated by replicating the results on two subsamples consisting of 690 individuals.

The third step was to calculate linear distances between manually identified 37 landmarks. Additional distances were included by automatically calculating the position of 14 mid-points of bilateral landmarks. These 51 landmarks determined 1275 linear distances (51x50/2). However, only 1247 of them show different results. The reason is that the 14 pairs of bilateral landmarks and their respective mid-points produce 42 distances, with only 14 of them being independent. For instance, distances ‘alare left’–‘alare right’, ‘alare left’–‘mid-alare’, and ‘alare right’–‘mid-alare’ are not independent.

The intraclass correlation coefficients, r_MZ_ and r_DZ_, were calculated for each facial parameter for 1208 twins who had a pair (263 MZ and 341 DZ). These formed the bases for further analyses on genetic and environmental contribution to facial morphology, as explained below.

### Assumptions and equations of the classical twin model

The classical twin study design relies on the following assumptions: 1) MZ twins share almost 100% of their genetic material; 2) DZ twins share about 50% of their genes and 3) MZ and DZ twins share the same common environment [45,46]. For each trait, the total phenotypic variance is assumed to be the sum of the variances due to genetic contribution, common environmental contribution and unique environmental contribution. Accordingly, the relative contributions due to heritability (h^2^), common environment (c^2^) and unique environment (e^2^) add up to one: h^2^ + c^2^ + e^2^ = 1. Gene-environment interactions are neglected in the model. The scanning and landmarking errors (1% and 5% respectively; see ‘error estimates‘) can be included into e^2^, so that the unique environment component is actually e^2^ less total error (6%). The correlation (similarity) between MZ twins is a product of shared genes (broad-sense heritability, h^2^) and c^2^. Broad-sense heritability involves both additive and non-additive genetic influences, as well as the epistasis [7]. Therefore, the intraclass correlation coefficient for MZ twins equals r_MZ_ = h^2^ + c^2^. The intraclass correlation coefficient for DZ twins equals r_DZ_ = 0.5h^2^ + c^2^. The unique environment and errors can be evaluated from the equation: e^2^ = 1 − r_MZ_. The common environment component can be evaluated as c^2^ = 2r_DZ_ − r_MZ_; if the result is positive, it indicates that common environment contributes to the facial trait variation. Negative values of 2r_DZ_ − r_MZ_ would imply a possible dominant genetic effect.

### Error estimates

To estimate the variance due to errors in scanning and landmarking, a sample of 73 faces (5% of the original database sample) was randomly selected for an intra-examiner reliability analysis. The faces were landmarked on two occasions six months apart by placing 37 landmark points. The reliability of a landmark was evaluated as the mean 3D Euclidean distance between its positions determined at two landmarking sessions.

For rough estimates, it was assumed that all the landmarks were independent. The total variance of landmark coordinates was evaluated as the sum of variances of all individual coordinates across the sample. After Procrustes registration of either set of 73 landmark configurations, the variances of the 111 individual coordinates were calculated. The total variance (V) for one set was 519.1 mm^2^ and 485.9 mm^2^ for the other, with the mean equal to 502.5 mm^2^.

According to Lübbers et al. [36], the mean global error of a 3dMD stereophotogrammetry system was 0.2 mm for a mannequin head. For live subjects, this should be at least doubled. The total variance due to scanning errors (V_SE_) was calculated as 37 x 0.4^2^ = 5.9 mm^2^, which makes up about 1% (5.9/502.5) of the total variance.

The variance due to landmarking errors can be estimated as follows. In the reliability sample, each landmark was placed twice. If x_1_ and x_2_ are two measurements of the x-coordinate of a landmark, its true value and the error are estimated as (x_1_+x_2_)/2 and |x_1_−x_2_|/2, respectively. By summing up the variances of the individual errors of all coordinates, we get the variance due to landmarking errors (V_LE_) equal to 25.1 mm^2^, which makes up about 5% (25.1/502.5) of the total variance.

### Statistical analyses

The intraclass correlation coefficients with the corresponding *p*-values and 95% confidence intervals for all facial parameters (principal components and linear distances) of 1208 twins (263 MZ and 341 DZ pairs) were evaluated using a non-parametric approach implemented in R programs [47]. A bootstrapping technique and a resampling technique with 100,000 random permutations each were used for the calculations [48]. The level of statistical significance was set at 0.05.

## Results

The intra-examiner reliability for 31 out of 37 landmarks was less than 2 mm (Table 2). Six remaining landmarks had reliability between 3.0 and 6.4 mm. The sample size was adequate and all the landmarks were used for the subsequent analyses.

**Table 2:** Intra-examiner reliability results for 37 facial anthropometric landmarks from two landmarking sessions undertaken with a time interval of six months.

Landmark (abbreviation)	Mean distance (mm)	SD (mm)
**Glabella (g)**	1.54	1.03
**Nasion (n)**	1.69	1.23
**Endocanthion left (enl)**	1.11	0.74
**Endocanthion right (enr)**	1.25	0.66
**Exocanthion left (exl)**	1.44	0.82
**Exocanthion right (exr)**	1.17	0.77
**Palpebrale superius left (psl)**	1.29	0.74
**Palpebrale superius right (psr)**	1.18	0.77
**Palpebrale inferius left (pil)**	0.91	0.42
**Palpebrale inferius right (pir)**	0.89	0.48
**Pronasale (prn)**	1.13	0.66
**Subnasale (sn)**	1.12	0.79
**Alare left (all)**	1.23	0.86
**Alare right (alr)**	1.36	0.70
**Labiale superius (ls)**	0.63	0.34
**Labiale inferius (li)**	0.84	0.51
**Crista philtri left (cphl)**	0.95	0.62
**Crista philtri right (cphr)**	0.84	0.49
**Cheilion left (chl)**	1.37	1.05
**Cheilion right (chr)**	1.11	0.63
**Pogonion (pg)**	3.00	1.44
**Gnathion (gn)**	2.98	1.83
**Zygion left (zyl)**	6.08	3.05
**Zygion right (zyr)**	6.43	3.28
**Subalare left (sbal)**	1.46	0.75
**Subalare right (sbar)**	1.43	0.77
**Philtrum nasale left (phnl)**	1.26	0.81
**Philtrum nasale right (phnr)**	1.03	0.66
**Mid-philtrum left (mphl)**	0.92	0.55
**Mid-philtrum right (mphr)**	0.83	0.49
**Deepest point of philtrum (dpc)**	0.64	0.42
**Mid-upper lip left (mull)**	1.15	0.80
**Mid-upper lip right (mulr)**	1.05	0.70
**Mid-lower lip left (mlll)**	1.11	0.72
**Mid-lower lip right (mllr)**	0.89	0.59
**Gonion left (gol)**	6.36	3.68
**Gonion right (gor)**	5.85	3.14

Note: See Fig 1 and Table 1 for description of landmarks.

**Fig 2:**
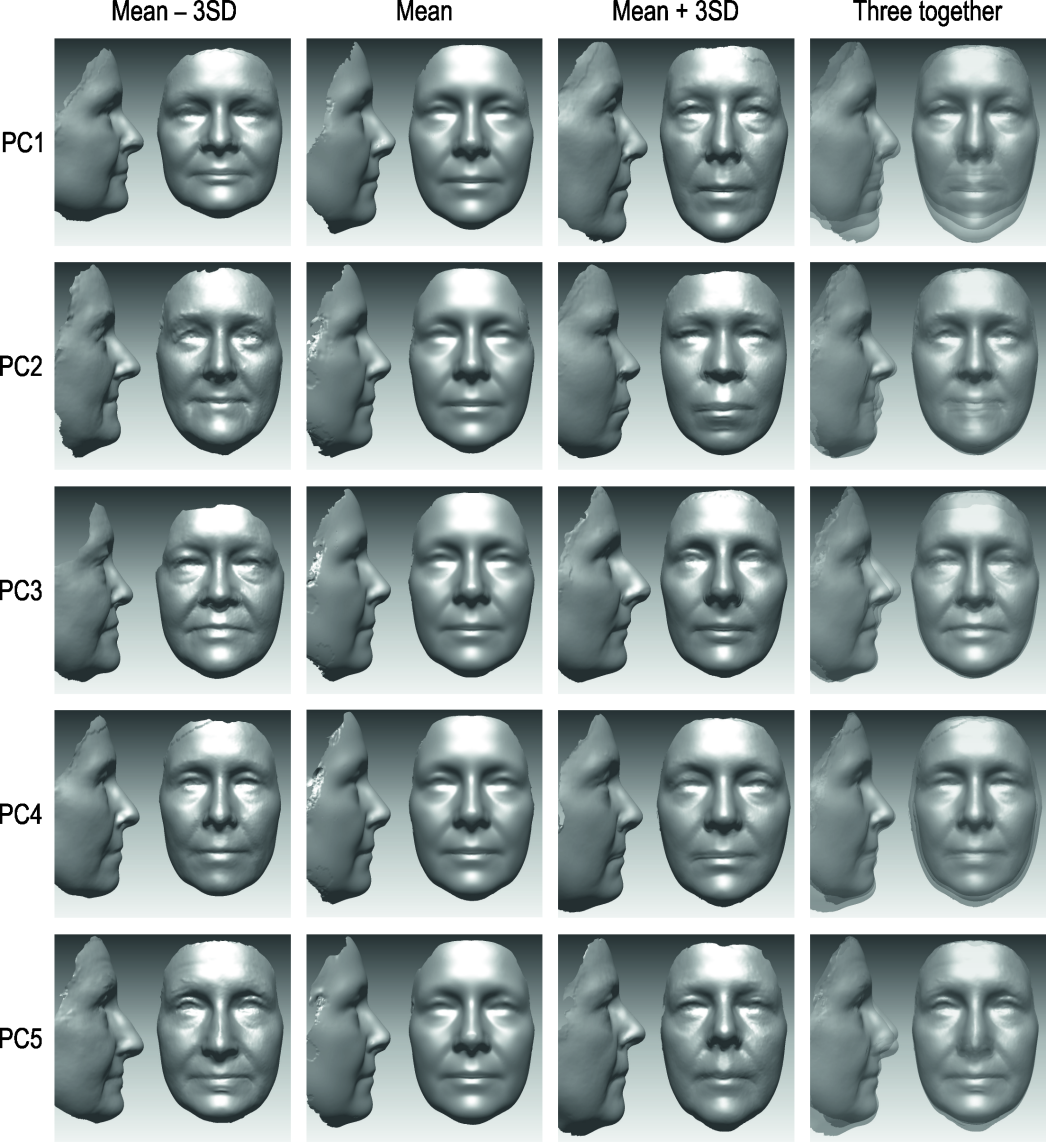
The effects of the first five unscaled principal components on the face. The average faces shown correspond to mean PC score and mean PC score ± 3 standard deviations. The averaging was performed for the faces with PC score within ‘mean ± 0.5 SD’ as well as ‘< mean −2.5 SD’ and ‘> mean + 2.5 SD’, respectively.

The findings of the principal component analysis for the total sample were replicated in both subsamples (S1 Table). Nineteen uPC (accounting for 87% of the total variance in facial form) were found for the unscaled dataset and 23 sPC (accounting for 83% of the total variance in facial shape) were found for the scaled dataset (Table 3). Although each principal component was contributed by all 111 coordinates, there were only relatively few that showed significant contribution (S1 Table). The anatomical interpretation of the above principal components (Table 4) was based on only those coordinates that contributed the most (factor loadings over 0.5 in magnitude). The effect of a PC on the face may be illustrated using a sequence of average faces [27] corresponding to different PC scores (Fig 2).

**Table 3:** Principal components and their variances for the total sample and two subsamples.

	Total sample (N = 1380)		Subsample 1 (N = 690)		Subsample 2 (N = 690)	
	Variance explained		Variance explained		Variance explained	
	Individual %	Cumulative %	Individual %	Cumulative %	Individual %	Cumulative %
**uPC1**	20.310	20.310	20.766	20.766	20.103	20.103
**uPC2**	10.848	31.159	10.778	31.544	11.280	31.383
**uPC3**	10.094	41.252	10.325	41.869	9.960	41.344
**uPC4**	6.183	47.435	6.458	48.327	6.474	47.818
**uPC5**	5.907	53.342	5.628	53.955	6.180	53.998
**uPC6**	5.513	58.855	5.110	59.066	5.424	59.422
**uPC7**	4.639	63.494	4.444	63.509	4.532	63.954
**uPC8**	3.787	67.281	3.567	67.076	3.838	67.793
**uPC9**	3.270	70.551	3.181	70.257	3.361	71.154
**uPC10**	2.613	73.164	2.696	72.953	2.495	73.649
**uPC11**	2.216	75.380	2.304	75.256	2.269	75.918
**uPC12**	2.072	77.452	2.093	77.349	2.119	78.037
**uPC13**	1.909	79.361	1.940	79.290	1.866	79.903
**uPC14**	1.715	81.076	1.750	81.040	1.675	81.577
**uPC15**	1.533	82.609	1.543	82.582	1.417	82.994
**uPC16**	1.197	83.806	1.248	83.830	1.189	84.183
**uPC17**	1.176	84.982	1.230	85.061	1.121	85.304
**uPC18**	1.089	86.071	1.122	86.182	1.040	86.344
**uPC19**	0.982	87.054	1.018	87.200	0.979	87.323
**sPC1**	14.870	14.870	15.613	15.613	14.267	14.267
**sPC2**	9.911	24.781	9.730	25.343	10.317	24.584
**sPC3**	8.886	33.668	8.798	34.141	8.988	33.572
**sPC4**	5.741	39.409	5.837	39.978	5.799	39.371
**sPC5**	5.428	44.837	5.562	45.540	5.414	44.785
**sPC6**	4.627	49.464	4.941	50.481	4.341	49.126
**sPC7**	3.960	53.424	3.858	54.339	4.125	53.251
**sPC8**	3.556	56.979	3.433	57.772	3.663	56.914
**sPC9**	3.172	60.151	2.991	60.763	3.324	60.238
**sPC10**	2.870	63.021	2.836	63.598	2.931	63.169
**sPC11**	2.392	65.413	2.390	65.988	2.435	65.603
**sPC12**	2.150	67.563	2.269	68.257	2.200	67.803
**sPC13**	2.098	69.661	2.071	70.327	2.064	69.867
**sPC14**	1.868	71.529	1.817	72.145	1.906	71.773
**sPC15**	1.704	73.233	1.659	73.804	1.795	73.569
**sPC16**	1.598	74.831	1.533	75.337	1.563	75.132
**sPC17**	1.453	76.284	1.422	76.759	1.459	76.591
**sPC18**	1.318	77.602	1.376	78.135	1.419	78.010
**sPC19**	1.250	78.851	1.220	79.355	1.290	79.300
**sPC20**	1.221	80.072	1.195	80.550	1.160	80.459
**sPC21**	1.121	81.193	1.134	81.684	1.049	81.509
**sPC22**	0.970	82.162	1.014	82.698	0.949	82.458
**sPC23**	0.915	83.078	0.923	83.621	0.883	83.341

PC, principal component; u, unscaled; s, scaled.

**Table 4:** Approximate description of facial principal components.

	Description		Description
**uPC1**	Facial size (height)	**sPC1**	Prominence of the upper lip relative to the chin
**uPC2**	Prominence of the lips	**sPC2**	Eyes height, width of the zygoma, width of the body of the mandible and vertical position of the lower lip and the chin
**uPC3**	Prominence of the nose	**sPC3**	Prominence of the nose
**uPC4**	Inter-ocular distance	**sPC4**	Philtrum height
**uPC5**	Nose height	**sPC5**	Upper lip height
**uPC6**	Ratio of the width of the lips and commissures’ depth	**sPC6**	Inter-ocular distance
**uPC7**	Upper lip height	**sPC7**	Ratio of the width of the lips and commissures’ depth
**uPC8**	Ratio of the depth of the eyes and zygomas	**sPC8**	Horizontal asymmetry of the base of the nose
**uPC9**	Width of the base of the nose	**sPC9**	Nose height
**uPC10**	Horizontal asymmetry of the nose, philtrum and lips	**sPC10**	Nose width
**uPC11**	Vertical position of the zygoma	**sPC11**	Horizontal asymmetry of the lips
**uPC12**	Prominence of the nasal root	**sPC12**	Vertical position of the zygoma
**uPC13**	Horizontal asymmetry of the nasal root, lips and chin	**sPC13**	Vertical position of the nasal root
**uPC14**	Width of the body of the mandible	**sPC14**	Philtrum width
**uPC15**	Prominence of the chin relative to mandibular angle	**sPC15**	Prominence of the lower lip
**uPC16**	Vertical position of the nasal root	**sPC16**	Ratio of the depth of the zygoma and depth of the eyes
**uPC17**	Facial width (left to right zygoma)	**sPC17**	Prominence of the nasal root
**uPC18**	Vertical position of the angle of the mandible	**sPC18**	Horizontal asymmetry of the chin
**uPC19**	Sagittal position of zygoma and mandibular angle	**sPC19**	Vertical position of the angle of the mandible
		**sPC20**	Horizontal asymmetry of the nasal root
		**sPC21**	Sagittal position of the angle of the mandible
		**sPC22**	Depth of the outer corners of the eyes
		**sPC23**	Vertical position of the corners of mouth relative to the vertical dimension of the chin

PC, principal component; u, unscaled; s, scaled.

Genetic and environmental contributions to facial principal components are presented in Table 5. Broad-sense heritability (h^2^) was statistically significant for majority of principal components: 13 out of 19 uPC (ranging from 38.8% to 78.5%) and 17 out of 23 sPC (ranging from 30.5% to 84.8%). Heritability of 50% and above was found for 12 uPC and 10 sPC. Morphology of the nose (width, height and prominence), prominence of the lips, inter-ocular distance and facial size had the highest heritability (all above 70%). Common environment significantly contributed to the variation in uPC11 and sPC12 (vertical position of the zygoma). Potential dominant genetic effect was found for sPC1 (prominence of the upper lip relative to the chin). The contribution of unique environment was statistically significant for 17 out of 19 uPC (ranging from 23% to 83%) and 20 out of 23 sPC (ranging from 24.6% to 87.7%). The highest unique environment contribution was found for uPC13 (horizontal asymmetry of the nasal root, lips and chin), uPC19 (representing sagittal position of zygoma and mandibular angle) and uPC10 (horizontal asymmetry of the nose, philtrum and lips). High unique environment contributions of over 50% was also found for uPC18, uPC16 and uPC11. These principal components were associated with the vertical position of the zygoma and the mandibular angle (could be interpreted as mandibular ramus height) and vertical position of the landmark ‘nasion’ (i.e., nasal root). In the scaled dataset, these corresponded to sPC8, sPC11, sPC18 and sPC20.

**Table 5:** Genetic and environmental contributions to facial principal components.

Principal components	r_MZ_	r_DZ_	h^2^ = 2(r_MZ_ − r_DZ_)	c^2^ = 2r_DZ_−r_MZ_	e^2^ = 1 –r_MZ_
**Unscaled dataset**					
**uPC1**	0.758 (0.703, 0.802)	0.380 (0.290, 0.462)	**0.756 (0.559, 0.954)**	0.002 (-0.181, 0.175)	0.242 (0.198, 0.297)
**uPC2**	0.680 (0.611, 0.737)	0.288 (0.182, 0.385)	**0.783 (0.544, 1.026)**	-0.103 (-0.324, 0.104)	0.320 (0.263, 0.389)
**uPC3**	0.688 (0.620, 0.745)	0.328 (0.218, 0.427)	**0.721 (0.481, 0.968)**	-0.033 (-0.259, 0.177)	0.312 (0.255, 0.380)
**uPC4**	0.770 (0.709, 0.819)	0.384 (0.292, 0.468)	**0.772 (0.565, 0.981)**	-0.001 (-0.192, 0.176)	0.230 (0.181, 0.291)
**uPC5**	0.645 (0.552, 0.721)	0.284 (0.181, 0.379)	**0.722 (0.459, 0.979)**	-0.077 (-0.296, 0.133)	0.355 (0.279, 0.448)
**uPC6**	0.629 (0.557, 0.691)	0.369 (0.273, 0.456)	0.520 (0.293, 0.747)	0.109 (-0.092, 0.299)	0.371 (0.309, 0.443)
**uPC7**	0.718 (0.654, 0.771)	0.383 (0.264, 0.489)	0.669 (0.421, 0.927)	0.049 (-0.193, 0.271)	0.282 (0.229, 0.346)
**uPC8**	0.578 (0.486, 0.656)	0.265 (0.160, 0.362)	0.625 (0.359, 0.887)	-0.048 (-0.271, 0.167)	0.422 (0.344, 0.514)
**uPC9**	0.565 (0.474, 0.643)	0.173 (0.063, 0.275)	**0.785 (0.511, 1.054)**	-0.219 (-0.452, 0.005)	0.435 (0.357, 0.526)
**uPC10**	0.170 (0.041, 0.288)	0.031 (-0.083, 0.141)	0.279 (-0.061, 0.607)	-0.109 (-0.364, 0.148)	**0.830 (0.712, 0.959)**
**uPC11**	0.440 (0.328, 0.539)	0.417 (0.324, 0.501)	0.046 (-0.234, 0.316)	**0.394 (0.186, 0.597)**	**0.560 (0.461, 0.672)**
**uPC12**	0.743 (0.678, 0.794)	0.358 (0.260, 0.447)	**0.769 (0.549, 0.990)**	-0.026 (-0.228, 0.165)	0.257 (0.206, 0.322)
**uPC13**	0.096 (-0.023, 0.212)	-0.018 (-0.131, 0.093)	0.228 (-0.098, 0.550)	-0.132 (-0.383, 0.122)	**0.904 (0.788, 1.023)**
**uPC14**	0.580 (0.484, 0.660)	0.248 (0.139, 0.349)	0.662 (0.386, 0.936)	-0.083 (-0.317, 0.141)	0.420 (0.340, 0.516)
**uPC15**	0.603 (0.515, 0.676)	0.322 (0.216, 0.421)	0.561 (0.298, 0.818)	0.041 (-0.182, 0.258)	0.397 (0.324, 0.485)
**uPC16**	0.394 (0.278, 0.497)	0.260 (0.161, 0.351)	0.268 (-0.025, 0.557)	0.126 (-0.099, 0.343)	**0.606 (0.503, 0.722)**
**uPC17**	0.550 (0.453, 0.632)	0.356 (0.253, 0.449)	0.388 (0.117, 0.650)	0.163 (-0.059, 0.374)	0.450 (0.368, 0.547)
**uPC18**	0.343 (0.225, 0.452)	0.271 (0.170, 0.363)	0.144 (-0.156, 0.440)	0.200 (-0.029, 0.419)	**0.657 (0.548, 0.775)**
**uPC19**	0.100 (-0.025, 0.219)	0.087 (-0.021, 0.191)	0.026 (-0.299, 0.345)	0.074 (-0.172, 0.316)	**0.900 (0.781, 1.025)**
**Scaled dataset**					
**sPC1**	0.703 (0.635, 0.759)	0.196 (0.076, 0.306)	**1.015 (0.755, 1.279)**	**-0.312 (-0.557, -0.079)**	0.297 (0.241, 0.365)
**sPC2**	0.638 (0.555, 0.705)	0.359 (0.270, 0.440)	0.557 (0.328, 0.782)	0.081 (-0.110, 0.262)	0.362 (0.295, 0.445)
**sPC3**	0.749 (0.691, 0.795)	0.344 (0.239, 0.439)	**0.808 (0.585, 1.038)**	-0.060 (-0.275, 0.140)	0.251 (0.205, 0.309)
**sPC4**	0.644 (0.563, 0.711)	0.345 (0.243, 0.436)	0.597 (0.356, 0.841)	0.046 (-0.167, 0.245)	0.356 (0.289, 0.437)
**sPC5**	0.692 (0.619, 0.752)	0.386 (0.265, 0.495)	0.611 (0.349, 0.880)	0.081 (-0.166, 0.310)	0.308 (0.248, 0.381)
**sPC6**	0.695 (0.616, 0.759)	0.271 (0.167, 0.366)	**0.848 (0.603, 1.093)**	-0.153 (-0.370, 0.053)	0.305 (0.241, 0.384)
**sPC7**	0.595 (0.518, 0.662)	0.293 (0.193, 0.383)	0.604 (0.364, 0.845)	-0.009 (-0.220, 0.189)	0.405 (0.338, 0.482)
**sPC8**	0.089 (-0.048, 0.220)	-0.049 (-0.159, 0.060)	0.275 (-0.073, 0.616)	-0.186 (-0.440, 0.071)	**0.911 (0.780, 1.048)**
**sPC9**	0.640 (0.551, 0.714)	0.318 (0.211, 0.414)	0.644 (0.382, 0.903)	-0.004 (-0.229, 0.209)	0.360 (0.286, 0.449)
**sPC10**	0.711 (0.635, 0.771)	0.323 (0.228, 0.409)	**0.777 (0.547, 1.001)**	-0.065 (-0.264, 0.124)	0.289 (0.229, 0.365)
**sPC11**	0.123 (0.002, 0.239)	-0.017 (-0.131, 0.097)	0.281 (-0.054, 0.605)	-0.157 (-0.414, 0.102)	**0.877 (0.761, 0.998)**
**sPC12**	0.467 (0.360, 0.558)	0.388 (0.296, 0.472)	0.156 (-0.113, 0.416)	**0.310 (0.105, 0.509)**	0.533 (0.442, 0.640)
**sPC13**	0.425 (0.313, 0.524)	0.320 (0.219, 0.410)	0.211 (-0.080, 0.496)	0.214 (-0.011, 0.428)	0.575 (0.476, 0.687)
**sPC14**	0.301 (0.186, 0.402)	0.108 (0.007, 0.207)	0.386 (0.081, 0.674)	-0.085 (-0.311, 0.146)	0.699 (0.598, 0.814)
**sPC15**	0.446 (0.346, 0.534)	0.254 (0.151, 0.350)	0.385 (0.107, 0.656)	0.061 (-0.161, 0.279)	0.554 (0.466, 0.654)
**sPC16**	0.474 (0.372, 0.563)	0.271 (0.171, 0.363)	0.405 (0.131, 0.672)	0.069 (-0.148, 0.280)	0.526 (0.437, 0.628)
**sPC17**	0.754 (0.689, 0.805)	0.371 (0.275, 0.457)	**0.765 (0.549, 0.982)**	-0.011 (-0.208, 0.174)	0.246 (0.195, 0.311)
**sPC18**	-0.069 (-0.176, 0.035)	0.039 (-0.062, 0.136)	-0.216 (-0.504, 0.074)	0.147 (-0.078, 0.369)	**1.069 (0.965, 1.176)**
**sPC19**	0.370 (0.255, 0.476)	0.230 (0.128, 0.323)	0.281 (-0.015, 0.575)	0.089 (-0.140, 0.308)	0.630 (0.524, 0.745)
**sPC20**	-0.018 (-0.134, 0.097)	0.061 (-0.037, 0.155)	-0.158 (-0.457, 0.144)	0.140 (-0.087, 0.362)	**1.018 (0.903, 1.134)**
**sPC21**	0.551 (0.451, 0.635)	0.306 (0.195, 0.408)	0.491 (0.205, 0.768)	0.060 (-0.175, 0.290)	0.449 (0.365, 0.549)
**sPC22**	0.485 (0.383, 0.573)	0.333 (0.241, 0.418)	0.305 (0.040, 0.559)	0.180 (-0.023, 0.379)	0.515 (0.427, 0.617)
**sPC23**	0.406 (0.302, 0.498)	0.214 (0.111, 0.309)	0.385 (0.103, 0.660)	0.021 (-0.202, 0.240)	0.594 (0.502, 0.698)

PC, principal component; u, unscaled; s, scaled; r_MZ_ and r_DZ_, intra-class correlation coefficient in MZ and DZ twins respectively; h^2^, broad-sense heritability; c^2^, relative common environment contribution; e^2^, relative unique environment contribution. Note: The figures in bold indicate heritability (h^2^) above 70%; statistically significant contributions of common environment (positive in the unscaled and negative in the scaled dataset) and statistically significant contributions of unique environment (above 70%) to facial principal components.

The genetic and environmental contributions to facial linear distances are presented in S2 Table. Thirty most heritable distances are presented in Table 6. Statistically significant genetic contribution was found for 1222 out of 1247 investigated independent distances. Broad-sense heritability of 70% and above was found for 476 distances (38.2%) and a heritability of 90% and above for 41 (3.3%) distances.

**Table 6:** Genetic and environmental contributions to thirty most heritable facial linear distances.

Linear distances	r_MZ_	r_DZ_	h^2^ = 2(r_MZ_ − r_DZ_)	c^2^ = 2r_DZ_−r_MZ_	e^2^ = 1 –r_MZ_
**prn-mex**	0.808 (0.758, 0.848)	0.285 (0.180, 0.379)	1.048 (0.832, 1.271)	-0.239 (-0.452, -0.041)	0.192 (0.152, 0.242)
**prn-men**	0.842 (0.802, 0.874)	0.341 (0.238, 0.434)	1.002 (0.798, 1.217)	-0.160 (-0.368, 0.032)	0.158 (0.126, 0.198)
**pir-prn**	0.778 (0.729, 0.817)	0.280 (0.174, 0.375)	0.997 (0.782, 1.220)	-0.219 (-0.431, -0.021)	0.222 (0.183, 0.271)
**exr-prn**	0.688 (0.613, 0.747)	0.192 (0.089, 0.288)	0.992 (0.749, 1.228)	-0.304 (-0.517, -0.098)	0.312 (0.253, 0.387)
**prn-mpi**	0.854 (0.817, 0.884)	0.363 (0.270, 0.447)	0.982 (0.796, 1.176)	-0.127 (-0.316, 0.046)	0.146 (0.116, 0.183)
**psr-prn**	0.730 (0.662, 0.785)	0.242 (0.137, 0.338)	0.977 (0.741, 1.213)	-0.246 (-0.462, -0.042)	0.270 (0.215, 0.338)
**mphl-men**	0.796 (0.753, 0.832)	0.311 (0.214, 0.399)	0.971 (0.775, 1.176)	-0.175 (-0.371, 0.008)	0.204 (0.168, 0.247)
**sn-men**	0.801 (0.752, 0.841)	0.319 (0.225, 0.405)	0.964 (0.765, 1.169)	-0.163 (-0.355, 0.017)	0.199 (0.159, 0.248)
**enr-prn**	0.793 (0.746, 0.831)	0.313 (0.204, 0.412)	0.958 (0.739, 1.190)	-0.166 (-0.389, 0.038)	0.207 (0.169, 0.254)
**phnl-men**	0.796 (0.748, 0.835)	0.318 (0.225, 0.403)	0.956 (0.759, 1.157)	-0.159 (-0.349, 0.019)	0.204 (0.165, 0.252)
**mphl-mex**	0.773 (0.719, 0.816)	0.296 (0.196, 0.387)	0.953 (0.741, 1.172)	-0.181 (-0.385, 0.010)	0.227 (0.184, 0.281)
**dpc-men**	0.795 (0.752, 0.831)	0.319 (0.222, 0.407)	0.953 (0.756, 1.158)	-0.158 (-0.353, 0.025)	0.205 (0.169, 0.248)
**men-mmph**	0.799 (0.757, 0.834)	0.323 (0.226, 0.411)	0.953 (0.757, 1.157)	-0.154 (-0.350, 0.028)	0.201 (0.166, 0.243)
**enl-prn**	0.822 (0.776, 0.858)	0.347 (0.248, 0.437)	0.949 (0.747, 1.160)	-0.127 (-0.329, 0.058)	0.178 (0.142, 0.224)
**men-mphn**	0.801 (0.753, 0.840)	0.329 (0.235, 0.414)	0.944 (0.747, 1.147)	-0.143 (-0.334, 0.035)	0.199 (0.160, 0.247)
**phnl-mex**	0.776 (0.719, 0.820)	0.304 (0.207, 0.393)	0.943 (0.732, 1.155)	-0.167 (-0.365, 0.020)	0.224 (0.180, 0.281)
**mphl-mpi**	0.806 (0.760, 0.843)	0.336 (0.241, 0.422)	0.940 (0.745, 1.143)	-0.134 (-0.326, 0.045)	0.194 (0.157, 0.240)
**prn-mps**	0.772 (0.709, 0.822)	0.304 (0.202, 0.397)	0.936 (0.710, 1.164)	-0.164 (-0.374, 0.033)	0.228 (0.178, 0.291)
**mex-mmph**	0.776 (0.724, 0.819)	0.309 (0.208, 0.400)	0.935 (0.725, 1.152)	-0.159 (-0.363, 0.032)	0.224 (0.181, 0.276)
**mphl-msbal**	0.686 (0.594, 0.758)	0.219 (0.105, 0.323)	0.935 (0.659, 1.204)	-0.249 (-0.487, -0.021)	0.314 (0.242, 0.406)
**mex-mphn**	0.782 (0.727, 0.825)	0.315 (0.218, 0.404)	0.933 (0.724, 1.145)	-0.151 (-0.349, 0.035)	0.218 (0.175, 0.273)
**dpc-mex**	0.773 (0.720, 0.816)	0.307 (0.206, 0.399)	0.933 (0.719, 1.152)	-0.160 (-0.365, 0.033)	0.227 (0.184, 0.280)
**mphr-men**	0.799 (0.758, 0.833)	0.336 (0.239, 0.423)	0.927 (0.733, 1.130)	-0.128 (-0.324, 0.053)	0.201 (0.167, 0.242)
**sn-mpi**	0.806 (0.758, 0.845)	0.343 (0.250, 0.426)	0.927 (0.733, 1.125)	-0.121 (-0.307, 0.055)	0.194 (0.155, 0.242)
**mpi-mmph**	0.808 (0.763, 0.845)	0.346 (0.252, 0.432)	0.924 (0.730, 1.126)	-0.116 (-0.307, 0.063)	0.192 (0.155, 0.237)
**dpc-mpi**	0.806 (0.760, 0.844)	0.345 (0.250, 0.431)	0.923 (0.727, 1.127)	-0.117 (-0.309, 0.063)	0.194 (0.156, 0.240)
**cphl-men**	0.800 (0.754, 0.837)	0.339 (0.240, 0.428)	0.923 (0.722, 1.131)	-0.122 (-0.321, 0.062)	0.200 (0.163, 0.246)
**sn-mex**	0.765 (0.704, 0.814)	0.305 (0.209, 0.393)	0.921 (0.707, 1.135)	-0.156 (-0.352, 0.032)	0.235 (0.186, 0.296)
**enl-mphl**	0.775 (0.726, 0.815)	0.315 (0.217, 0.404)	0.921 (0.717, 1.132)	-0.146 (-0.345, 0.040)	0.225 (0.185, 0.274)
**phnr-men**	0.798 (0.747, 0.838)	0.337 (0.242, 0.423)	0.921 (0.721, 1.126)	-0.123 (-0.317, 0.056)	0.202 (0.162, 0.253)

r_MZ_ and r_DZ_, intra-class correlation coefficient in MZ and DZ twins respectively; h^2^, broad-sense heritability; c^2^, relative common environment contribution; e^2^, relative unique environment contribution. S2 Table contains calculations for all investigated linear distances.

Common environment was found to have a significant positive contribution to 53 out of 1247 distances (4.3%). These distances are between: 1) bilateral landmarks ‘zygion’ and different landmarks located on the tip of the nose, lips, mandibular angle (landmark ‘gonion’) and chin and 2) eye and lip landmarks and middle points between bilateral ‘zygion’ and ‘gonion’.

Possible dominant genetic effect was found for 11 distances (0.9%). These were: three distances between right eye landmarks and the tip of the nose (‘prn’); one distance between the bilateral ‘exocanthion’ midpoint and the tip of the nose (‘prn’); two distances between the tip of the nose and two nose base landmarks (‘sn’, ‘phnr’); two distances between the most prominent point of the chin (‘pg’) and two lower lip landmarks (‘li’, ‘mmll’) and three distances between upper lip philtrum points (‘mphl’, ‘mphlr’ and ‘dpc’) and nose base points (‘sbalr’, ‘msbal’). These distances represent the prominence and height of the nose, prominence of the lower lip in relation to the chin and length of the upper lip philtrum. The contribution of unique environment was significant in 1245 out of 1247 distances.

## Discussion

Phenotypic variation in humans is produced through a complex interplay between genotype and environment, but the characterization of phenotype lags behind the characterization of the genotype [49]. In facial research, there is no uniform approach to the analysis of facial phenotype due to: 1) variable facial data acquisition (two-dimensional or three-dimensional techniques); 2) lack of a standardized way to quantify spurious expressions (relying on subjective opinion of the examiner); 3) variable selection and/or definition of phenotypic traits; 4) the amount of information analysed (sparse or spatially-dense points across the facial surface that can be identified manually or automatically) and 5) statistical analyses (univariate/multivariate).

In this study, facial phenotype was characterized by principal components and linear distances based on 37 anthropometric landmarks manually identified on the 3D facial images. Most of the landmarks could be reliably identified (within 2 mm), except for soft tissue ‘zygion’ (difficult to describe in anatomical terms and traditionally identified by trial measurement), ‘gonion’ (normally identified by palpation), ‘pogonion’ and ‘gnathion’ (these were difficult to identify in vertical plane if a chin had a flat surface) [39]. However, as the sample was quite large (1380 twin faces), it was decided to include these landmarks in the subsequent analyses.

We found that soft tissue facial traits in adult British female twins have moderate to high heritability, which is in general agreement with previous family and twin studies. In Koreans [6], the heritability values ranged from 0.25 (lower facial height, sn-gn) to 0.61 (intercanthal width; en-en) for linear measurements derived from digital photographs of family members; the authors also identified three factors with heritability estimates from 0.45 (total face height, upper face height and nose height) to 0.55 (lower face height and width of the mandible, mouth and nose). High correlations between parents and offspring and siblings were also found in Indian families [50] for mandibular position, chin prominence, nasal prominence, nasal width, lip length at philtrum, lip prominence and facial height. However, heritability was not calculated. In Belgian families [51], heritability estimates for soft tissue facial parameters ranged from 0.46 (nose height) to 0.72 (external biocular breadth). Cephalometric analysis of soft tissue parameters in Turkish siblings [21] revealed high heritability (0.72 to 1.0) for soft tissue chin thickness, soft tissue facial angle, Merrifield angle (formed by the Frankfort plane and profile line joining the chin and the more prominent lip, usually the upper) and Holdaway angle (formed by a line tangent to the chin and upper lip with the cephalometric NB line). Differences in estimates can be explained by different methodology: photos [6, 50, 51] or lateral cephalograms [21]; measurement errors; small samples and different ethnicities. In addition, the components of genetic variance for a given trait vary from population to population [51].

The first 3D twin study focusing on soft tissues [28] was performed on British twins (10 pairs of MZ and 8 pairs of same-sex DZ twins aged 9 to 17 years) using stereophotogrammetry. Significant differences between faces of MZ and DZ twins were found for intercanthal width, right eye width, nose width, nose height, mouth width, and upper lip height. This coincides with our results, despite the obvious differences in the sample size and age. In [29], faces of British twins (10 pairs of MZ and 10 pairs of same-sex DZ twins) were also analysed using laser scanning. It was apparently the first time that facial surfaces were suggested for analysis apart from analyzing linear distances. Significant genetic determination was revealed for midfacial parameters, especially left eye width, intercanthal width, nose height, and nose width. However, the study by Naini and Moss [29] did not show any significant differences in mouth width and upper lip height, which contradicts the study done by Burke [28] as well as our findings, which show a strong genetic contribution above 60% for these traits. The differences can be explained by the small sample used in [29], wide age range of participants (6–42 years) and their mixed ethnicity, as well as no formal heritability calculation.

In [38], a well-known British population study (ALSPAC) was the source of 37 twin pairs, whose faces were captured using laser scanning. Configurations of 21 facial landmarks as well as facial surfaces were compared between 19 MZ and 18 DZ twin pairs aged 15 years. Procrustes analysis did not reveal any significant difference in facial landmark configurations. On the other hand, average female MZ and DZ twin faces coincided in the eyes, supraorbital and infraorbital ridges, philtrum and lower part of the cheeks. In the absences of heritability estimates, the findings of that study indirectly show that central facial structures are the most heritable ones.

A preliminary study performed on American twins [32], 10 MZ and 11 same-sex DZ twins 5–12 years of age, found that only three out of nine extracted principal components showed statistically significant genetic contribution. These were related to the horizontal separation between the eyes, the length, breadth and projection of the nose, and the height and projection of the upper lip. Heritability estimates approached 1.0 and the authors explained this over-estimation by the small sample size, small number of landmarks and a very crude calculation of heritability. In our study, a significantly greater number of landmarks were used and therefore more principal components were extracted. Most of these components showed an evidence of statistically significant genetic contribution.

Various landmark-based traits (e.g.,distances, angles, ratios and principal components) as well as surface-based traits (e.g., geodesic distances and curvatures) can be used to seek genes responsible for normal facial morphological variation. Only a few genome-wide association studies have been conducted so far, which have revealed a relatively small number of associations between certain facial traits and single-nucleotide polymorphisms (SNPs). The findings are in agreement with our results on highly heritable traits, especially those associated with the morphology of the nose and lips. The SNPs found in the *PAX3* gene [52–54] are associated with the nasal root morphology in Europeans and Latin Americans. In addition, multiple intronic SNPs in the *PRDM16* gene are associated with nose width and nose height [53]. A SNP close to the *C5orf50* gene is associated with the position of the landmark ‘nasion’[53]. An intronic SNP in *TP63* gene is associated with the inter-ocular distance and a missense SNP in *COL17A1* is associated with the distance between the eyes and ‘nasion’ [53]. Significant associations were recently found for three more nose-related traits: columella inclination (4q31), nose bridge breadth (6p21) and nose wing breadth (7p13 and 20p11) [54]. The rs642961 SNP in the *IRF6* gene (a known risk factor of non-syndromic cleft lip/palate) was found to strongly predict normal lip shape variation in Han Chinese females but not males [55].

The finding that facial asymmetry is not genetically driven complies with the results of two studies, [38] and [56]. In the first one, the amount of three-dimensional asymmetry was calculated after superimposing (registering) the original face with its mirror reflection and measuring the average distances between the two facial surfaces. There was no statistically significant difference in the amount of facial asymmetry between MZ and DZ twins [38]. In the other study, the relationship between facial asymmetry (evaluated from nine mid-facial landmarks) and genetic variation at 102 SNP loci (recently associated with facial shape variation) was investigated. The authors failed to identify any SNP relating to either fluctuating or total asymmetry [56].

Our finding on the mandibular ramus height is in agreement with a recent cephalometric study conducted on 141 adult Lithuanian twin pairs with completed mandibular growth and DNA confirmed zygosity [57]. The authors estimated the significance of additive (A) and non-additive (D) genetic factors as well as shared (C) and unique environment (E) using a maximum likelihood genetic structural equation. Their results indicate that the shape and sagittal position of the mandible is under stronger genetic control than is its size and vertical relationship to cranial base. For linear measurements, such as mandibular body length, ramus width and ramus height, the best-fitting model was found to be ACE, indicating low genetic determination.

The present study has certain advantages and limitations, which will be discussed below. One of the merits is a large sample size, which enabled us to use additional landmarks (in comparison to previous studies on facial morphology) and contributed to the validity of heritability estimates (without compromising statistical power). In addition, the sample was homogenous in terms of ethnicity and came from a well-designed population-based study. Finally, zygosity was confirmed by genetic testing, hence avoiding the misclassification of twins.

On the other hand, the limitations of the study were due to: 1) limitations of the classical twin design, 2) complexity of facial morphology and 3) inclusion of only female individuals in the sample. The observed facial morphological variation is a combination of: 1) genetic contribution (encompassing additive and non-additive genetic effects as well as gene interactions), 2) environmental contribution (consisting of common and unique environment), 3) gene-environment interaction and 4) measurement errors (due to scanning and landmarking).

The trait correlation between twins is the result of their genetic similarity and sharing common environment. However, the classical twin design does not allow for the determination of any gene-environment interactions. In addition, the scanning and landmarking errors (usually included in the unique environment component) may have some effect (generally negligible) on the heritability values of some traits, especially those that just reach statistical significance (*p*-values less than but close to 0.05). Genetic contributions calculated here are likely to be slightly overestimated because the model disregards gene-environment interactions. Furthermore, the imperfections of the classical twin model can lead to heritability values (h^2^) over 1, which are demonstrated by the following results: the heritability (h^2^) of sPC1 was evaluated as 1.015 (95%CI: 0.755 to 1.279), h^2^ of the linear distance ‘prn-men’ was 1.002 (95%CI: 0.798 to 1.217) and h^2^ of the ‘prn-mex’ distance was 1.048 (95%CI: 0.832 to 1.271). However, these do not diminish the importance of our findings. Instead of focusing on the actual value of an estimate, it is more important to reveal which facial traits demonstrate the most compelling evidence of heritability and use that knowledge for future genome-wide association studies of normal facial morphology.

The second limitation is related to the complexity of studying facial morphology (as explained in the introductory paragraph of the discussion). The third limitation is due to inclusion of only females in the sample. This reflects the prevalence of females in the TwinsUK register [34]. In the data available to us, there were 15 males, which were excluded intentionally as they were too few for a separate statistical analysis; the inclusion of male individuals in a predominantly female sample could have affected the findings. This issue needs to be addressed in future studies. It would be interesting to look at a sufficiently large male twin sample and compare the results with those for female twins. In addition, the sample we dealt with was ethnic specific and further research is needed on other populations, since environmental effects and gene alleles frequencies may differ between populations [51,52].

## Conclusions

The study provides the estimates of genetic and environmental contributions to three groups of landmark-based facial traits in adult female twins. Based on the analysis of principal components, statistically significant genetic influence on the facial form was found to range from 38.8% to 78.5%, whereas that on the facial shape accounts for 30.5% to 84.8% of the total phenotypic variance. Genetic factors can explain more than 70% of the phenotypic variance in 7 principal components related to facial form, 5 principal components related to facial shape and 474 linear distances. These facial parameters represent: facial size (height), nose (width, prominence and height), lips prominence and inter-ocular distance. A few traits show potential dominant genetic influence, namely the prominence and height of the nose, the prominence of the lower lip in relation to the chin and length of the upper lip philtrum. The highly heritable traits are likely candidates for genome-wide association studies. Environmental contribution to facial variation is the greatest in the mandibular ramus height and horizontal facial asymmetry. This heritability study may inform future genetic studies which facial traits should be focused on.

## Supporting Information

S1 Table The results of the Principal Component Analysis (PCA) for the total sample and two sub-samples (unscaled and scaled datasets). (XLSX)

S2 Table Relative genetic and environmental contributions to facial linear distances. (XLSX)
