# Correction: Genetic and Environmental Contributions to Facial Morphological Variation: A 3D Population-Based Twin Study

**DOI:** 10.1371/journal.pone.0164961

**Published:** 2016-10-12

**Authors:** The PLOS ONE staff

The byline designation for the Visigen Consortium is incorrect. The correct name is: International Visible Trait Genetics (Visigen) Consortium, and the members can be found in the Acknowledgments section. The publisher apologizes for the error.
